# Supplementary material for: Are carbon emissions trading and green financial instruments synergistic? -Comprehensive quantitative research based on content analysis
Source: PLoS One. 2024 Mar 7;19(3):e0298601. doi: 10.1371/journal.pone.0298601 (PMC10919601; doi:10.1371/journal.pone.0298601)
Supplement: S1 Data — (ZIP) [file pone.0298601.s001.zip › Data/地级市Z_F工作报告.zip!/╕╩╦α/╝╬╙°╣╪╩╨/└┤╫╘╣1⁄2╓┌║┼í╛┬φ┐╦╩2╛▌═°í┐_2013.docx]

各位代表：

　　现在，我代表市人民政府向大会作工作报告，请予审议，并请各位政协委员和列席人员提出意见和建议。

　　2012年工作回顾

　　今年以来，在省委、省政府和市委的坚强领导下，在市人大、市政协的监督支持下，全市人民深入贯彻科学发展观，紧紧围绕转型跨越、争先进位的要求，牢牢把握“稳中求进、好中求快”的总基调，积极应对复杂多变的经济环境，迎难而上，努力进取，圆满完成了九届人大一次会议确定的各项任务。预计实现地区生产总值251.8亿元，增长24.3%（同口径现价比）；规模以上工业增加值170亿元，增长20%；地区性财政收入36.7亿元，增长12.7%；市级财政收入15.1亿元，增长20%；完成固定资产投资94亿元，增长44.2%；城镇居民人均可支配收入22170元，增长17.1%；农村居民人均纯收入10980元，增长18.1%。

　　工业结构调整取得突破。深入推进工业强市战略，大力培育战略性新兴产业。酒钢（集团）公司嘉酒风电基地载能特色铝合金节能技术改造项目2×45万吨电解铝、不锈钢冷轧二期、2×350兆瓦自备电厂项目开工建设，年销售收入突破1000亿元大关，位列中国企业500强第128位。新能源及装备制造业发展迅速，嘉西百万千瓦光伏产业园获批装机容量663兆瓦，已建成和在建334兆瓦。甘肃广银一期45万吨铝制品深加工、索通公司一期25万吨预焙阳极、大友公司一期4万吨优质碳黑等项目顺利投产。甘肃嘉峪关经济技术开发区正式挂牌，全年预计实现工业总产值180亿元，首次跨入全省百亿元开发区行列。加大政银企项目融资对接力度，全市金融机构贷款余额预计达到290亿元，增长26.6%。

　　会展经济快速发展。成功举办第二届敦煌行·丝绸之路国际旅游节开幕式及系列活动、第八届国际铁人三项赛、第四届国际航空滑翔节、首届国际文化产业大会暨第五届甘肃省文博会、首届国际短片电影展等一批重大节会活动，促进了文化、旅游、体育、商贸服务业的融合发展，有力提升了城市影响力和美誉度。组建了文化旅游投资（集团）有限公司，中华孔雀苑获评国家3A级景区，评定31家星级农家乐，成功入围全国第二批旅游标准化试点城市。东方百盛购物中心、新河西商贸城改扩建项目投入使用，酒钢新世纪广场启动招商运营。全市旅游人数突破280万人次，增长27%；旅游及相关产业收入达到16.1亿元，增长33%；全市文化产业完成投资3.7亿元，增长5倍，实现增加值2.3亿元，增长25%；社会消费品零售总额达到37.6亿元，增长25.4%。

　　人居环境继续改善。坚持新城区开发和旧城区改造并重，进一步优化城市功能布局，加强生态环境建设，群众生活更加舒适便捷。新区道路、供热、通讯等基础设施不断完善，讨赖河生态环境治理三、四期工程全部完成，“两湖一河”园林景观、龙王滩湿地公园建设进展顺利，新增水面23.6万平方米。完成了关城景区周边、嘉酒城际一级公路两侧等六项重点绿化工程，新增绿地面积316.8万平方米。实施了城市环境综合整治，改造了昌盛、友谊等6个居民小区和兰新东路、阳关路等11条城市道路。狠抓清洁生产、节能降耗和污染减排，主要污染物排放均控制在目标范围内。

　　城乡一体化建设稳步推进。相继完成了农村土地整理、环境连片整治、饮用水源保护、农产品质量安全检验监测站建设、镇村文化室改造等项目。新建集中连片日光温室145座、移动式钢架大棚200座，支持养殖小区建设13个。现代农业生态示范园、文殊戈壁日光温室基地等农业产业化项目进展顺利。新成立专业合作社40个，农业产销组织化程度明显提高。扶持农村居民创业致富，由政府贴息500万元，发放农村妇女小额担保贷款4856万元。“联村联户、为民富民”行动取得阶段性成效，为农村居民办实事400多件，密切了干群关系，促进了农村发展。

　　社会事业均衡发展。坚持科教兴市，实施国家级科技项目9项，省级科技项目11项，市级科技项目87项，科技对经济社会发展的支撑能力日益增强。“两基”攻坚任务全面落实，教育教学条件明显改善，各类教育协调发展，教育质量稳步提高。医药卫生体制改革深入推进，市精神卫生中心主体工程完工，基层医疗服务网络进一步完善。文化强市战略深入实施，世界文化遗产保护和展示工程进展顺利，组建了长城文化和丝路文化研究会，书香雄关、唱响雄关等“十个雄关”文化活动深入开展，农村广播电视“户户通”工程在全省率先完成。全国全民健身示范城市创建工作扎实开展，新华、胜利等4个社区体育健身中心顺利建成。全面推行了公交公营改革，收购两家民营公司公交车91辆，新增新能源公交车15辆，为市民创造了更加安全、舒适、便捷的乘车环境。成功创建全国双拥模范城“五连冠”。工商、质监、税务、粮食、供销、电力、邮政、通信、人防、统计、档案、地方志、气象、地震、防灾减灾、民族宗教、外事侨务、科普、国防教育、人口与计划生育、妇女儿童、老龄、慈善等工作也都取得了新的进展。

　　民生保障力度加大。认真落实就业政策，积极促进高校毕业生就业，多渠道开发就业岗位，全年新增城镇就业8057人，城镇登记失业率控制在了2.7%以内。不断加大劳动监察工作力度，农民工权益得到切实保护。社会保障体系不断健全，解决了6388名“五七工”、“家属工”养老保障历史遗留问题，城乡居民社会养老保险参保率达到95%，城镇基本医疗保险参保率达到99%，新型农村合作医疗参合率达到 98.4%，工伤保险在全市机关事业单位全面展开。大幅提高城乡低保标准和五保供养标准，低保标准高出全省指导线40.4%，五保供养标准高出2倍，保障水平全省最高。

　　在全面完成省政府14件实事的基础上，市政府承诺的10件惠民实事全部完成：（1）建成了10个“农超对接”平价店和15个市场物价监测点，建立了价格监测预警和市级生猪、蔬菜储备制度，居民消费价格指数控制在103%以内；（2）建成了150套、7500平方米的廉租住房；（3）市一中、市六中D级危房拆除重建顺利推进，市一幼、市二幼拆除重建和市五幼、市六幼、三镇中心幼儿园新建项目全部完成并投入使用；（4）改造旧城区给排水、供热等管网30.5公里，对昌盛、友谊、兰新、雍和、五一、祁连等6个居民小区进行了美化、绿化、亮化改造；（5）为所有农村居民点安装太阳能路灯2200盏，实施了5个污水处理和1个农村饮用水水源保护项目，全市17个农村社区垃圾收集清运设施全部配备到位；（6）在市区建设公益性停车场7个，改造原有停车场4个，新增停车泊位651个；（7）新图书馆购置图书10万册，原图书馆改造为少儿图书馆，市文化馆、图书馆、三镇综合文化站等公益性场馆全部免费开放；（8）新建及改造城市公共卫生间8座，市政管理的18座公共卫生间全部免费开放；（9）对全市残疾人参加基本养老、医疗、工伤、失业、生育保险给予补助，受益2695人次；（10）为全市35-59岁妇女免费进行妇女病普查的任务顺利完成。

　　社会管理创新形成特色。被中央政法委确定为全国9个社会管理创新典型培育城市之一，经验得到了中央和省委、省政府的充分肯定，并在全国交流推广。实施了城乡户籍一元化、社会管理扁平化、“平安和谐细胞”等“三大工程”，建成了社会管理服务、城乡一体化服务和社会矛盾大调解“三大中心”。撤销街道并组建了30个新型城市社区，对17个行政村实行了社区化管理，培育了6个城乡标准化示范社区，配套创建了流动人口、“两新”组织等6个方面的服务管理机制，形成了具有嘉峪关特色的社会管理服务体系。开通了“12345”民生服务热线，整合社保、民政、卫生等公共服务资源，为11000多名60岁以上老人免费配备了“一键通”呼叫终端，提供政府补贴的亲情式居家养老服务，初步建成了政府引导、社会化运作、企业服务的社区居家养老服务模式。社会治安管控、信访等工作扎实推进，突发事件应急管理机制不断完善，生产、交通、校园、食品药品等安全监管工作进一步加强，社会更加和谐稳定。

　　开放合作步伐加快。嘉酒城际一级公路建成通车，区域内深层次、全方位的合作交流和经贸往来不断加强，加快了经济一体化进程。新开通了嘉峪关至深圳航线，进一步完善了航线网络布局，形成了与环渤海、长三角、珠三角、成渝等经济发达地区的联系通道，航空运输旅客吞吐量突破30万人次，同比增长35.1%。将“走出去”和“引进来”相结合，与安徽、浙江、江苏、广东等10多个省的100多家知名企业进行广泛洽谈，与华强集团、正大集团成功达成项目合作协议。第十八届“兰洽会”签约项目24个，签约总金额175.9亿元。全年招商引资到位资金62.5亿元，增长68.6%。

　　政府建设不断加强。民主法治建设持续推进，依法行政水平不断提高。自觉接受人大依法监督和政协民主监督，全年共办理人大建议89件、政协提案77件。政府决策和信息发布等制度进一步健全，重大事项社会听证、公示和专家咨询制度得到较好落实。设立了“市长信箱”，建立了“市长接待日”制度，解决了一大批群众关注的热点、难点问题。深入推进效能风暴行动，成立了政务服务中心，清理并取消企业筹建登记等行政审批项目39项。认真落实政府廉政建设责任制，强化行政监察和审计监督，加强经营性土地使用权出让、工程招投标、政府集中采购等重点领域的监督检查，实行了政府投资项目“代建制”管理，为从源头上遏制腐败建立了制度保障。

　　各位代表，过去的一年，在复杂严峻的国际国内经济形势下，全市各级干部和广大人民群众付出了艰辛努力，做了大量卓有成效的工作，经济社会发展呈现出了稳中有进的良好态势，实现了新一届政府工作的良好开局。这些成绩的取得，是省委、省政府和市委坚强领导的结果，是市人大、市政协有力监督和全力支持的结果，是嘉峪关军分区、酒钢（集团）公司、中核四○四有限公司及驻嘉各单位共同努力的结果，是全市上下解放思想、齐心协力、开拓进取的结果。在此，我代表市人民政府，向全市各族人民，向人大代表、政协委员，向驻嘉部队、武警和消防官兵、政法干警，向各民主党派、工商联、无党派人士、人民团体、社会各界人士和离退休老同志表示崇高的敬意和衷心的感谢！

　　在看到成绩的同时，我们也清醒地认识到，全市经济社会发展中还存在一些突出的矛盾和问题，主要表现在：一是经济总量偏小、结构性矛盾突出，仍然是当前和今后全市经济社会发展中的主要问题；二是资源、环境等发展要素制约逐步加大，节能减排任务艰巨；三是群众关心的公共医疗卫生设施和服务等一些问题还没有得到很好的解决；四是政府自身建设还需不断强化，推动科学发展的能力需要进一步提升。这些问题，既是影响经济社会发展的难点，也是我们下一步工作的重点和着力点，我们将高度重视并采取有效措施，认真加以解决。

　　2013年主要工作

　　2013年，是我市调整经济结构、转变发展方式，实施“十二五”规划承上启下的关键之年，促发展、保增长、惠民生的任务十分繁重。我们要切实增强发展意识，坚定发展信心，抢抓国家扩大内需、调整优化产业布局、促进经济发展方式转变的新机遇，借势而行，乘势而上，努力保持良好的发展势头，推进全市经济社会平稳较快发展。

　　政府工作的总体思路是：深入学习和全面贯彻落实党的十八大、省第十二次党代会、市第十次党代会精神，牢牢把握科学发展、转型跨越、富民强市这个主题，紧紧抓住转变经济发展方式这条主线，按照中央和全省经济工作会议的部署，更加注重产业结构调整、经济总量扩容、城乡统筹发展、城市品位提升、民生事业改善和社会和谐稳定，努力推动经济社会率先转型跨越发展，加快建设现代化区域中心城市。

　　经济社会发展的主要预期目标是：地区生产总值增长25%（现价），规模以上工业增加值增长20%，全社会固定资产投资增长45%，地区性财政收入增长20%，市级财政收入增长20%，社会消费品零售总额增长25%，城乡居民收入增长16%，城镇登记失业率控制在2.8%以内，人口自然增长率控制在6.5‰以内，单位生产总值能耗和污染物排放完成控制指标。

　　围绕上述目标，要重点做好以下十个方面的工作：

　　一、立足市情，明确方向，努力实现项目建设新跨越

　　坚持把项目建设作为推动经济社会转型跨越发展的重中之重，认真贯彻落实省委、省政府实施“3341”项目工程总体部署，实施好我市“4661”项目工程，着力打造以百亿元园区为载体的经济发展平台、以文化旅游深度融合为牵引的第三产业发展平台、以新区开发为重点的城市发展平台、以和谐幸福城市为目标的社会建设平台“四个平台”，瞄准工业转型升级、生态环境保护、文化旅游融合、城乡协调发展、保障改善民生、区域联动开发“六个主攻方向”，紧紧围绕优质钢铁、现代高载能循环经济示范、新能源示范、优质食品加工、长城文化与丝路文化传承创新发展、区域商贸物流“六大基地”建设，加大项目筛选、储备、申报力度，积极争取国家和省上资金、政策支持，着力建设一批具有全局性、战略性的重大项目，为到2016年生产总值力争突破1000亿元夯实基础。进一步完善领导包抓、部门负责、地企联动的项目工作机制，加大考核奖惩力度，在全社会形成聚精会神抓项目、凝心聚力促发展的良好氛围。

　　二、扩大总量，优化结构，不断提高工业经济质量和效益

　　以循环经济示范市建设为抓手，大力实施优势主导产业提升、战略性新兴产业增量、工业园区扩容“三大行动”，着力优化产业结构。全力支持酒钢（集团）公司嘉北产业新区和循环经济及结构调整项目建设，做好嘉酒风电基地载能特色铝合金节能技术改造项目2×45万吨电解铝、2×350兆瓦自备电厂等重点项目的服务保障。加快推进嘉西光伏产业园建设，实施好中国华电、上海航天、中利腾晖等光伏发电项目，力争年内达到1200兆瓦建设规模。加快建设330千伏汇集站，切实解决清洁能源输送、消纳问题。全力推进广银铝业二期55万吨铝制品深加工、大友公司循环经济产业链等工业项目建设，支持甘肃申能、西部重工、大陆桥和海特等新能源装备制造企业加快发展。实施嘉北、嘉西供水扩容工程，加快经济技术开发区配套设施建设，盘活存量土地，构建产业集群发展平台，努力创建国家级经济技术开发区。支持企业科技创新，推动成果转化，鼓励引导企业与高等院校、科研院所开展技术合作，全市综合科技进步水平指数达到45%以上。全面落实“一产一策、一企一策”服务措施，抓好安全生产、节能降耗和污染物减排，为工业经济持续快速增长提供有力支撑。

　　三、节会带动，产业引领，促进文化旅游体育深度融合发展

　　以华夏文明传承保护和创新发展示范区建设为契机，坚持品牌节会带动、特色产业引领，不断促进文化、旅游、体育融合发展。加快推进嘉峪关世界文化遗产保护和展示工程建设，重点实施世界文化遗产监测中心、游客服务中心和长城博物馆新馆三大单体工程。高起点编制旅游发展总体规划，完善基础设施、提升服务水平、加大宣传促销力度，争创旅游标准化示范城市。抓好观礼古镇、游客集散综合服务中心等项目建设，适时启动首旅自驾游营地、七一冰川探险营地、讨赖河大峡谷省级地质公园、新城草湖国家级湿地公园等项目规划建设，构建立体化、多元化旅游产业链。规划建设文化旅游产业园区，做好产业布局规划，深化与国内外知名文化企业的洽谈合作，大力引进文化战略投资。支持文化旅游投资（集团）有限公司运用市场手段，实施项目融资和开发建设，推动文化旅游产业加快发展。大力培育发展会展经济，做好光伏产业博览体验中心和核科学与民用技术陈列馆规划筹建工作，积极申办2013年国际铁人三项赛、2014年铁人三项世界杯赛、第五届航空滑翔旅游节、全国汽车越野拉力赛，全力办好第三届敦煌行·丝绸之路国际旅游节系列活动和第二届国际短片电影展，倾力打造国际国内知名的品牌节会和精品赛事。

　　四、挖掘潜力，提升品质，加快建设休闲消费型城市

　　突出地域文化特色，科学制订城市休闲产业发展规划，完善基础设施，大力发展休闲消费产业。积极优化商贸流通行业布局，培育重点商贸流通企业。启动实施新华中路商贸中心、五一商城改造等项目，推动东方百盛购物中心、酒钢新世纪广场全面投入运营，加快形成新华路核心商贸聚集区。推进嘉峪关华强文化科技产业基地和华城置业城市综合体建设，启动龙王滩遗址公园水上舞台、仿古美食街项目，建成讨赖河大型音乐喷泉和水幕电影，引进星级酒店、水岸风情街等项目，着力完善城市休闲消费功能。进一步落实促进非公有制经济和中小企业发展的各项政策，切实发挥非公有制经济在休闲消费领域的主力军作用，促进交通、旅游、文化、餐饮、商贸、通信等产业快速发展。进一步加强政银企合作，努力扩大信贷投放，开发灵活多样的金融产品，着力提升金融支持经济社会发展水平。

　　五、完善功能，彰显特色，切实提高城市建设管理水平

　　坚持高起点规划、高标准建设、高效能管理，加快建设山水特色之城、生态园林之城、人文魅力之城。编制实施城市风貌和色彩规划，完善讨赖河两岸新区空间发展二期规划设计，把历史痕迹和文化符号融入城市规划建设，彰显地域特色。继续实施“两湖一河”园林景观工程、讨赖河生态环境治理和新区基础设施项目，加快新区建设步伐。启动雍平、建新街区改造，不断提升老城区服务功能。全力推进兰新铁路第二双线、火车南站、综合客运枢纽中心和嘉峪关至黑鹰山战备公路、金嘉公路嘉峪关段、西沟矿公路、长城旅游公路建设，改善综合通行和区域通行条件。实施酒钢新区、关城文化旅游景区南侧等重点绿化工程，对部分道路和居民小区进行绿化改造，新建绿地135.2万平方米，完成绿化改造面积20.4万平方米。对文化路改造、商业聚集区停车场等项目进行规划论证，适时启动改造建设。继续扩大公交规模，完善优化运营路线，方便群众出行。加大城乡环境卫生、市容市貌的整治管理力度，展现国家卫生城市新形象。加快推进数字雄关和智慧城市建设，建立健全网格化、智能化管理模式，不断提升城市管理水平。

　　六、加大投入，健全机制，深入推进城乡一体化进程

　　立足规划、政策、产业三个重点，加大统筹城乡发展力度，增强农村发展活力，促进城乡共同繁荣。完成安远沟、野麻湾、石桥、黄草营等4个试点村的新农村规划编制工作，启动农村新居试点建设，按照适度集中、人畜分离、产居分开的原则，科学布局住宅、生产、养殖区域，加快推进城乡基础设施一体化进程。继续实施一批交通、水利、土地整理、环境整治项目，建立健全农村公共设施管理和维护保障机制。大力发展特色优势产业，加快推进正大现代农业合作示范、现代农业生态示范园、观赏树种育苗等农业项目，引导龙头企业投资农业生产，促进农村土地加快流转。大力发展设施农业，新建改造三代日光温室100座，新建养殖小区5个。实施农村居民就业创业培训工程，完善农村金融服务体系，拓宽农业融资渠道。巩固和深化城乡户籍制度改革成果，强化农村公共服务，推动城乡就业、社保、教育、医疗加快实现均等化。扎实开展“联村联户、为民富民”行动，切实为农村居民解难题、办实事、谋发展。

　　七、巩固成果，拓展领域，全面提升社会管理科学化水平

　　深入推进全国社会管理创新样板市建设，推广试点经验，完善工作机制。加快标准化社区建设，完善全市各社区、各联动单位与“三大中心”的对接工作，不断提升民情流水线、12345民生服务热线等特色服务工作水平。完善社区考核机制，将为民服务水平作为社区工作考核的核心内容。进一步加强居家养老服务体系建设，建立健全信息网络和服务平台，促进居家养老服务标准化、规范化和集约化。深入推进平安嘉峪关建设，认真实施“平安和谐细胞”工程，继续完善城市视频监控网络。严格落实信访工作责任制，强化社会舆情应对机制，高度重视群体性事件防范和化解，切实维护社会和谐稳定。完善公共安全体系建设，全面加强食品、药品、交通和校园安全，有针对性地强化学校、医院、商场、机关等人流密集区域的突发灾害应急避险演练，提高应急救援和防灾避险能力，保障人民群众生命财产安全。

　　八、强化服务，保障民生，着力建设和谐幸福城市

　　多谋民生之利，多解民生之忧，在学有所教、劳有所得、病有所医、老有所养、住有所居上取得新进展。千方百计拓宽就业渠道，促进创业带动就业，推进收入倍增计划，切实增加城乡居民收入。全面实现教育均衡发展，巩固提高“两基”攻坚成果，实施中小学操场改造、农村学校危房加固改造工程，优化教育办学条件。把为人民群众提供更高水平的医疗卫生服务放在更加突出的位置，全面提升综合医院和专科医院服务能力，推进社区、农村医疗机构标准化建设。建立居民家庭健康档案，与高等院校合作培养社区全科医生30名，逐步形成综合医院、基层医疗机构和流动医院上门就诊相结合的医疗服务体系。继续拓展养老、医疗、工伤等社会保险覆盖面，巩固提高新农合保障水平和覆盖率。认真做好保障性住房建设，新建廉租房100套、公租房216套、经济适用房384套。全面加强人口和计划生育工作，促进人口长期均衡发展。加快完善基层文化设施，扶持和培育各类群众文化团队，广泛开展群众文化活动。加快推进全民健身示范城市建设，修建体育活动场地8000平方米，开展形式多样的全民健身活动。抓好市场供应和物价稳定，努力将居民消费价格指数控制在104%以内。制定出台更加务实的人才政策，培养实用人才，引进紧缺人才，为全市经济社会发展提供人才支撑。深入实施文明素质提升工程，积极创建全国文明城市。扎实开展全国双拥模范城“六连冠”创建活动。

　　2013年，重点办好以下惠民实事：（1）从3月1日起，我市60岁以上老人全部免费乘坐公交车；（2）对阳光、五一、迎宾二小区等3个居民小区进行环境综合改造；（3）实施嘉峪关市酒钢三中新校区、嘉峪关市四中综合教学楼二期工程建设，完成市一中、市六中教学楼改扩建工程并交付使用，完成5所中小学操场改造、6所农村中小学食堂建设项目；（4）启动新建市第一人民医院、妇幼保健院和儿童医院，改扩建市第二人民医院综合大楼、中医院和新城镇、文殊镇卫生院。为我市所有参合农民免费进行常规项目体检；（5）在全市开展居家养老日间护理上门服务；（6）建设城市公共自行车系统，在主要景点、宾馆、商场等地段设置7个服务站点，实行通借通还；（7）在人流密集的路段、公共场所安装3台智能图书借阅机和10个电子阅报栏；（8）实施农村中心敬老院、儿童福利院和社会福利院联建项目。建成残疾人康复托养中心；（9）新建改造城市标准化社区服务中心10个、农村标准化社区服务中心3个；（10）新建农超对接平价店10家，创建市级食品安全示范店25家；（11）在新老城区合理布局建设公共卫生间6座，购置生态公共卫生间4座，并免费开放；（12）建成政务服务中心和公共资源交易中心，实现“一厅式办公、一站式服务”。

　　九、深化改革，加强合作，进一步提升开放开发水平

　　深化改革开放，破解发展难题，营造充满活力的城市发展环境。继续深化城乡统筹、投融资、医药卫生、文化、水利等重点领域改革。积极推进嘉酒区域经济一体化，利用两地优势资源，促进要素流通、服务连通、产业融通，做大做强嘉酒经济圈。抢抓产业梯度转移机遇，围绕优质钢铁、有色冶金、新能源及装备制造、文化旅游、现代服务等产业，充分利用兰洽会、广交会等重要节会，建好承接平台，主动出击、积极衔接，强化区域招商。特别是瞄准世界500强、行业龙头企业，强化定点招商。开辟重大招商项目“绿色通道”，完善招商责任制度，提前介入、全程跟踪、强化服务，切实提高签约项目落实率。

　　十、改进作风，提高效能，努力建设阳光诚信政府

　　坚持依法行政，自觉接受人大依法监督、政协民主监督、新闻舆论监督和社会公众监督，进一步提高办理人大议案、代表建议和政协提案的质量。认真执行重大事项集体决策、专家咨询、社会公示与听证制度，深入实施政务公开、政府信息公开和公共企事业单位办事公开制度，积极推进网络问政，广纳群言、广集民智，使决策更加科学民主。扎实开展“六五”普法，全面加快依法治市进程。深入推进效能风暴行动，严格落实工作目标责任制，强化督查和跟踪问效，全面提升行政效能。加快政府职能转变，涉及行政审批和民生服务事项的有关部门全部进驻政务服务中心，进一步减少审批环节，简化办事程序，提高办事效率。认真落实中央关于改进工作作风、密切联系群众的八项规定，改进会风、文风，规范接待、警卫工作，厉行勤俭节约。严格落实廉政建设责任制，扎实推进惩治和预防腐败体系建设，从源头上遏制违纪、违法案件的发生。坚持开拓创新、真抓实干，说实话、办实事、求实效，努力建设人民信赖和满意的政府！

　　各位代表，转型跨越的重任催人奋进，富民强市的使命鼓舞人心。让我们在党的十八大精神指引下，在省委、省政府和市委的坚强领导下，坚定信心，凝心聚力，锐意进取，扎实工作，加快经济社会率先转型跨越发展，全面建设现代化区域中心城市，为创造全市人民更加幸福美好生活而努力奋斗！
